# Quantifying the value of on-farm measurements to inform the selection of key performance indicators for livestock production systems

**DOI:** 10.1038/s41598-021-96336-1

**Published:** 2021-08-19

**Authors:** Andy Jones, Taro Takahashi, Hannah Fleming, Bruce Griffith, Paul Harris, Michael Lee

**Affiliations:** 1 Rothamsted Research, North Wyke, Okehampton, EX20 2SB Devon UK; 2 University of Bristol, Langford, BS40 5DU Somerset UK; 3 Harper Adams University, Newport, TF10 8NB Shropshire UK

**Keywords:** Data integration, Systems analysis, Animal physiology

## Abstract

The use of key performance indicators (KPIs) to assist on-farm decision making has long been seen as a promising strategy to improve operational efficiency of agriculture. The potential benefit of KPIs, however, is heavily dependent on the economic relevance of the metrics used, and an overabundance of ambiguously defined KPIs in the livestock industry has disincentivised many farmers to collect information beyond a minimum requirement. Using high-resolution sheep production data from the North Wyke Farm Platform, a system-scale grazing trial in southwest United Kingdom, this paper proposes a novel framework to quantify the information values of industry recommended KPIs, with the ultimate aim of compiling a list of variables to measure and not to measure. The results demonstrated a substantial financial benefit associated with a careful selection of metrics, with top-ranked variables exhibiting up to 3.5 times the information value of those randomly chosen. When individual metrics were used in isolation, ewe weight at lambing had the greatest ability to predict the subsequent lamb value at slaughter, surpassing all mid-season measures representing the lamb’s own performance. When information from multiple metrics was combined to inform on-farm decisions, the peak benefit was observed under four metrics, with inclusion of variables beyond this point shown to be detrimental to farm profitability regardless of the combination selected. The framework developed herein is readily extendable to other livestock species, and with minimal modifications to arable and mixed agriculture as well.

## Introduction

Against the backdrop of rapid population growth and economic development, worldwide demand for animal source foods (ASF) continues to increase^1,2^. ASF play an important role in human nutrition as a source of high-quality protein and essential micronutrients, both of which are biologically difficult and economically costly to obtain from plant source foods alone^3–5^. However, agricultural systems to produce ASF are generally associated with lower land use efficiency compared to alternative land use^6^, making their areal expansion neither economically feasible nor socially desirable^7–9^. Increased demand for ASF therefore can only acceptably be met through improvements in land use efficiency of existing livestock systems^10–12^, or by filling the ‘yield gap’ between current production and the best potential production^13^. The presence of a substantial variability in production efficiency is widely recognised across the livestock industry^14^, even within systems operating under comparable climatic, biophysical and socioeconomic conditions^15^. Importantly, this is the case at both the farm scale^16^ and the animal scale^17^, with economic and environmental performances often positively correlated with one another regardless of the spatial resolution^18,19^. Thus, an effort to reduce the yield gap suffered by less efficient farm systems and less efficient animals are equally likely to enhance the industry’s capability for ASF provision.

As a means of decision support to facilitate this transformation, two interrelated frameworks have primarily been adopted in the farm management literature: benchmarking and identification of key performance indicators (KPIs). Of the two, the concept of benchmarking centres on a comparison of an individual farm’s performance against an externally defined standard, normally derived from a survey of comparable enterprises^20,21^. As such, this approach provides farms with a way to assess how efficiently their business is operating on a relative scale^22^. However, most benchmarking exercises take the form of whole business analysis based on aggregate measures rather than information arising from individual production processes, often resulting in output metrics that are not necessarily informative for day-to-day operation when used in isolation^23^. A 5-year study of pork enterprises in Iowa, US found that only 6% of sample farms were consistently ranked within the top-third in terms of profitability, while 67% were ranked in the bottom-third at least once^24^. This example demonstrates that an attempt to emulate exemplary on-farm practices from aggregated measures can be problematic, especially given that the method’s capability to identify the presence of an issue is not always accompanied by a solution^25^.

KPIs, on the other hand, are generally defined as variables closely related to production inputs, production outputs or production efficiency, selected with a higher-level goal of understanding the drivers behind an individual farm’s performance^26^. A study evaluating the Norwegian dairy sector employed a principal component analysis (PCA) to simultaneously identify financial and production factors contributing to gross margin, and then used this information to determine on-farm practices that should be promoted^27^. Another study in New Zealand quantified the level of resilience embedded into dairy farms through variables strongly associated with inter-farm variability, and from this information produced a list of target KPIs for low-performing farms to measure and thus improve^28^. In a study designed to determine KPIs for the income of Australian wool producers, the technical efficiency of farms was first estimated and then the data analysed through a PCA to identify production factors associated with maximum technical efficiency^29^. These farm-scale studies were explicitly designed to explore precision agriculture solutions for efficiency-related issues currently present within each flock/herd, thereby ultimately increasing the overall competitiveness of the local livestock industry.

The potential benefit of KPIs, however, is heavily dependent on the relevance of the variables to be used^20,27,30^. The number of livestock industry recommended KPIs has steadily increased since the agricultural intensification of the 1960s^31^, leading to a high level of duplication across a long list of variables^32^. This, in turn, has invited uncertainty around the exact purpose of KPI measurements, both in general and in particular to individual metrics, frequently resulting in a practically unconstructive message of ‘measure as much as you can’ without due comprehension of scientific rationales. Critically, on-farm performance monitoring requires considerable cost, time and resources^33^ yet offers no guarantee of benefit^22^; thus, such ambiguity around the meaning of KPIs can easily disincentivise farmers to collect any production data at all.

Using high-resolution sheep monitoring data from the North Wyke Farm Platform (NWFP), a system-scale grazing trial in Devon, UK^34^, this paper aims to develop a novel quantitative framework to evaluate the information value of various performance indicators on a livestock farm’s short-term economic performance. The UK sheep sector presents a unique and suitable case exemplar for the present study; despite its economic scale (£2.5 billion p.a.) and an extensive list of recommended KPIs made available to farmers^32^, it is known for an exceptionally low level of production performance monitoring^35^. In the past, this phenomenon has primarily been attributed to a heavy reliance on agricultural subsidy payments^36^, which reduces the need for in-depth analysis of on-farm income and expenditures^37^. However, the sector is predicted to be one of the most severely affected by the UK’s withdrawal from the European Union, and therefore improvement in productivity is urgently needed^38^.

Our case study will adopt end-of-season variables of slaughter age (days required to reach the target weight) and realised carcass value as short-term animal-level measures of economic performance. These variables represent the cost and revenue of the enterprise, respectively, and are known to be driving factors of UK sheep farms’ profitability^39–41^. The information value of a mid-season variable, or a performance indicator, will then be quantified in relation to the strength of its association with end-of-season measures and, based on this value, the relative usefulness of multiple indicators will be evaluated. The general framework has been designed to accommodate a wider range of performance indicators, for example at different spatial resolutions and from other livestock sectors, providing an evidence base to support farmers’ decisions on what to measure and what not to measure.

## Methods

### Use of experimental animals

All animal data used in this study were collected as part of standard farming practices. As such, no part of this research was subject to the Animals (Scientific Procedures) Act 1986 or approval of an ethics committee.

### Definitions of terminology

The aforementioned ambiguity about KPIs is likely to have stemmed, at least partially, from the fact that existing lists of variables indistinguishably include those that describe a farm’s enterprise structure, management strategies and performance, with no explicit recognition given to their interrelationships. To overcome this issue, variables commonly referred to as KPIs were first categorised into the following three groups prior to the quantitative analysis. As will be discussed, each group has a specific role in the subsequent computational process to calculate the redefined KPI values.

*Predictors* are defined as variables that do not directly represent the ultimate performance of the enterprise but are useful for its estimation. Akin to leading indicators in economics^42^, an example of a predictor is the 8 week weight of lambs; it does not equate to any financial value at the time of measurement but is strongly (although imperfectly) associated with finishing age which, in turn, affects production cost. Predictors are generally most useful for informing short-term decisions for adaptive farm management, for instance whether to provide supplementary feed, as this information can be collected before production of the final output.

*Outcomes*, on the other hand, are more directly linked to the ultimate performance of the enterprise, akin to lagging indicators in economics^43^. To continue the previous example, the finishing age of lambs can be seen as an outcome variable, as the causal relationship between this metric and profitability is almost certain. Unlike predictors, these variables are unhelpful for informing decisions about short-term changes, as the relevant information is collected after production is realised. They are, however, useful at long-term decision making across multiple seasons, as historic information in this form can be used to determine the optimal enterprise structure given the farm’s biophysical, financial and labour constraints.

The final category, *system descriptors*, is composed of variables that are frequently referred to as KPIs but more closely represent long-term strategic decisions taken by farm managers themselves. Ewe to ram ratio, for example, is often considered a KPI but is almost always a direct result of a human choice. Akin to diagnostic measures in economics^44^, system descriptors affect operation of the farm through multiple pathways and therefore likely have indirect impacts on its overall performance as well. However, they are of less importance as an indicator to assist adaptive decisions and should instead be seen as a set of constraints, or a rule of engagement, under which all other decisions are optimised in the short-term.

Based on the above definitions, KPIs currently in common usage by the livestock industry have been reclassified in Table 1. As discussed, the analytical framework proposed in this study was designed to select variables of which measurements should be prioritised to support a farm’s short-term decisions. In line with this goal, only *predictors* will be considered as performance indicators henceforth, with the view to identify those with high information values as redefined ‘key’ performance indicators vis-à-vis conventional ‘KPIs’. The information values of predictors will be quantified in relation to their capability to predict *outcomes* under a given set of *system descriptors*.

**Table 1:** Key performance indicators currently in common usage.

Indicator	Predictor	Outcome	Descriptor	Level applied	Current justification
Birth weight	X			Lamb	(Juengel et al., 2018)
Four-week weight	X			Lamb	(Wright, 2015)
Eight-week weight	X			Lamb	(Wright, 2015)
Weaning weight	X			Lamb	(EBLEX, 2014a)
Average daily liveweight gain	X			Lamb	(Gascoigne and Lovatt, 2015)
Slaughter age		X		Lamb	(Kerr, 2000)
Carcase conformation		X		Lamb	(Fisher and Heal, 2001)
Fat class		X		Lamb	(Fisher and Heal, 2001)
Kill-out percentage		X		Lamb	(Matthews and Ford, 2012)
Cold carcase weight		X		Lamb	(Stanford et al., 1998)
Body condition score	X			Ewe	(Kenyon et al., 2014)
Change in BCS	X			Ewe	(Kenyon et al., 2014)
Weight	X			Ewe	(Brown et al., 2015)
Weight change	X			Ewe	(Brown et al., 2015)
% lambs failing to reach 85% target weight	X			Farm	(Wright, 2018)
Ewe to Ram ratio			X	Farm	(EBLEX, 2008)
Scanning percentage	X			Farm	(Earle et al., 2016)
% empty ewes at scanning	X			Farm	(EBLEX, 2008)
Lambing percentage	X			Farm	(Morris, 2009)
Lambs alive after 48 h	X			Farm	(AHDB, 2015)
Lambs weaned	X			Farm	(Bohan et al., 2018)
Lambs reared		X		Farm	(AHDB, 2018)
Lamb losses from scanning to birth	X			Farm	(EBLEX, 2014a)
90 day lamb weight per ewe to ram	X			Farm	(AHDB, 2018)
Weight of lamb reared per ewe to ram		X		Farm	(EBLEX, 2014b)
Percentage of empty ewes	X			Farm	(EBLEX, 2008)
Ewe mortality	X			Farm	(EBLEX, 2014b)
Percentage of ewes culled			X	Farm	(EBLEX, 2008)
Flock replacement rate			X	Farm	(EBLEX, 2014b)

### Case study of the UK sheep sector

The case study was conducted at the NWFP in southwest UK (50º46′10″N, 3º54′05″W) over five grazing seasons between 2015 and 2019. The site has consistently high rainfall, characteristic of grassland regions of the UK, with a mean annual precipitation of 1030 mm over a 35-year period from 1984 to 2019. The interquartile ranges for daily minimum and maximum temperatures over the same period were 3.6–10.4 °C and 9.8–17.4 °C, respectively. The mean annual precipitation during the study period was 952 mm, whereas the interquartile ranges for daily minimum and maximum temperatures were 3.8–10.8 °C and 10.2–17.9 °C, respectively.

The NWFP consists of three self-contained enterprises locally known as ‘farmlets’, each adopting a different pasture-based grazing system typical of those found in temperate lowland grasslands (permanent pasture, reseeded grass monoculture and reseeded legume/grass mix)^45^. Sheep data collected for the present study encompassed all three farmlets, with the final dataset including 1364 lambs and their mother ewes (389 in total)^46^. The flock comprised Charollais rams and Suffolk x Mule ewes, producing an average of 2.01 lambs per year. Lambs were born indoors in March/April and turned out to pasture at 72 h postpartum. Ewes were housed pre-lambing and fed silage supplemented with concentrate feed; however once at pasture neither ewes nor lambs received any supplementary feed^47^. Ewes and lambs were initially placed on the same pasture and subsequently split into separate enclosures at weaning, which occurred at 90 days from the average lambing date. Lambs were screened for carcass quality (musculature and fat cover) upon reaching a target liveweight of ~ 40 kg via manual handling at the loin, dock, rib and breast, with those deemed expertly to meet the standard industry criteria separated for slaughter. Across five seasons, lambs were finished at an average of 177 days. Post-slaughter, information on cold carcass weight, carcass quality and current carcass price were obtained from the abattoir. These data were combined to compute the realised carcass value for each lamb and, as discussed above, employed as an *outcome* variable alongside the slaughter age.

In addition, 10 animal-level variables identified in Table 1 were collected as potential predictors. For lambs, liveweights were recorded at birth, 4 weeks, 8 weeks and 90 days (weaning). When the 4-week and 8-week measurements were not taken on the exact day, a linear adjustment was made to estimate the corresponding weight to ensure inter-animal comparability. For ewes, both bodyweight and body condition score (BCS) were measured at three key points during the production season, namely at lambing, weaning and tupping, with BCS graded manually^48^ by trained personnel.

**Figure 1:**
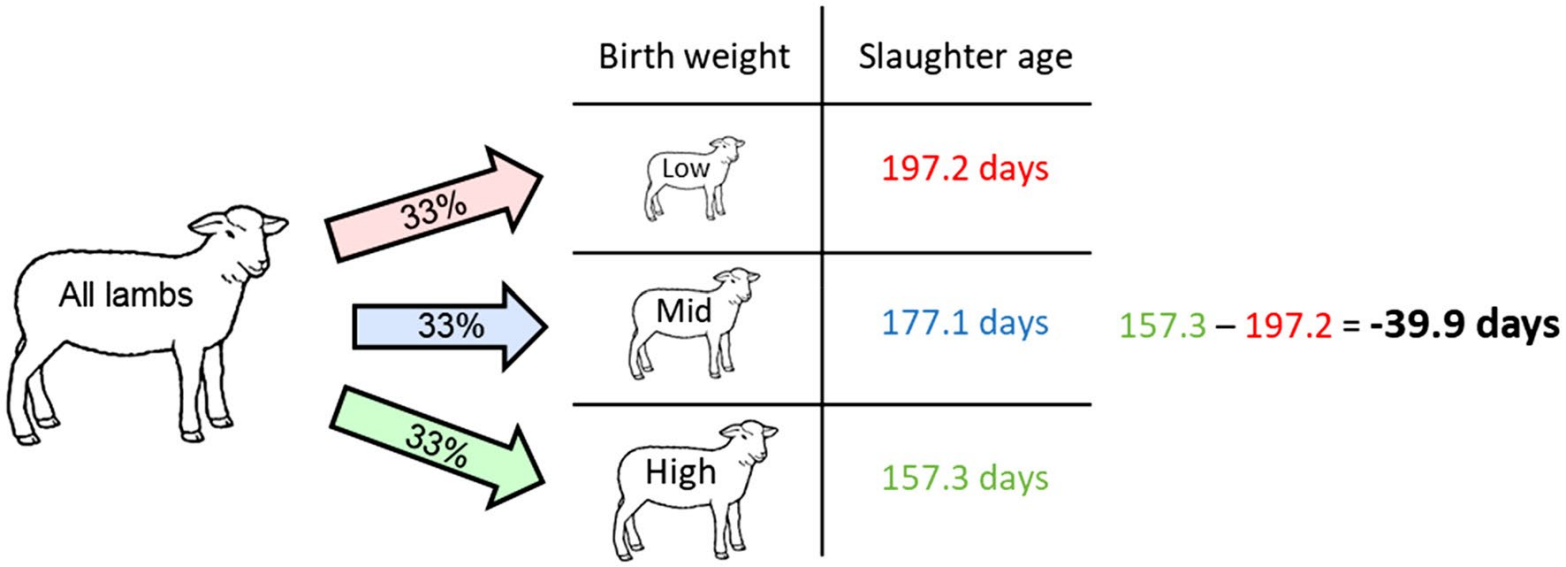
Proposed method to estimate the gross information value of a predictor. It is computed as the difference in end-of-season performance outcome (slaughter age in this example) between top (high) and bottom (low) groups, as defined mid-season according to the relevant predictor value (birth weight in this example). Top third and bottom third animals were allocated to ‘high’ and ‘low’ groups, respectively, for the baseline analysis. However, main results were insensitive to changes in how these two groups were defined. Produced by the authors using Microsoft PowerPoint.

Using this dataset, the gross information value of each predictor was defined by the potential benefit of employing adaptive management based on the said predictor value, as evaluated through the impact on the two outcome variables that are strongly associated with realised lamb sales and profit (defined above). Specifically, this information value was calculated in four stages (Fig. 1). Firstly, all lambs in the dataset were ordered according to the predictor value, for example according to their birth weight. Secondly, these lambs were divided into three equal-sized groups according to their rankings, for example top third (high), middle third (mid) and bottom third (low) groups according to their birth weight. Thirdly, the mean value for each outcome variable was obtained for each group, for example the average slaughter age of high, mid and low groups. Finally, the difference in this mean value between the high and low groups was calculated and statistically compared via *t*-test. The gross information value thus derived represents the expected economic benefit of an animal ‘upgrading’ from the low group to the high group according to each predictor, under the assumption that on-farm strategies exist to enable such manipulation.

It is worthwhile noting that the gross information value is exclusive of costs associated with data collection. The decision to use a gross value for the baseline analysis was taken to make the results applicable to a wider spectrum of sheep farms, as substantial variation in geographical conditions, and therefore labour and equipment costs, exists within the UK sheep sector. In other words, the gross value is more independent from the effect of the study site, and thus more directly representative of physiological mechanisms governing sheep performance. Notwithstanding, the implications of considering the cost of data collection will also be briefly investigated in the *Discussion* section.

The analysis outlined above is designed to evaluate the gross information value for each of 10 predictors individually. However, as many predictors are correlated with each other (Supplementary Tables S1 & S2), the benefit of using multiple predictors is not directly cumulative. Furthermore, as these correlations cause multicollinearity, the relative contribution of each predictor variable to the outcome variable cannot be quantified through standard regression models. To overcome these challenges, a nonparametric procedure was devised to estimate the combined gross information value of multiple predictors on carcass value.

Here, for each predefined number of predictors (1–10), the average ranks of individual lambs across multiple predictors were first calculated for all possible combinations of predictors. The number of mathematically possible combinations ranged from 1 (for 10 predictors, $\frac{10!}{1!\left(10-1\right)!}$) to 252 (for 5 predictors, $\frac{10!}{5!\left(10-5\right)!}$). Using this average ranking, the information value of the relevant combination was then estimated in a similar manner as the single predictor case described above. This resulted in a paired list matching predictors used for ranking and their collective information value. Intuitively, the *marginal* value of a predictor when added to a set of other variables depends on the covariance structure across the two groups, with a stronger association generally leading to a lower benefit due to information redundancy. Thus, the current approach is conceptually analogous to model selection processes commonly employed to identify the best regression model, albeit tailored to the situation where most variables are correlated with one another.

Finally, in order to appraise the sensitivity of the main findings to the definition of the high and low groups (top third and bottom third as evaluated by predictors), the entire procedure was repeated twice using alternative classification rules. In the first test the high and low groups were defined as equal halves (top half and bottom half); in the second test, they were defined as equal quarters (top quarter and bottom quarter).

All data analyses were conducted using R version 4.0.2^49^.

## Results

When slaughter age was used as the outcome variable, predictors directly linked to lamb weight had the highest information value. Weaning weight, 8-week weight and 4-week weight showed an average value of 84.9, 75.2 and 64.4 days (to slaughter), respectively (Table 2). Using carcass value as the outcome, predictors linked to ewe weight and BCS were more valuable than those linked to lamb weight, with ewe weight and BCS at lambing valued at £3.34 and £2.69, respectively. The discrepancy between the most informative (ewe weight at lambing) and the least informative (ewe weight at weaning) predictors was £2.35, demonstrating a substantial financial benefit to the appropriate selection of metrics.

**Table 2:**
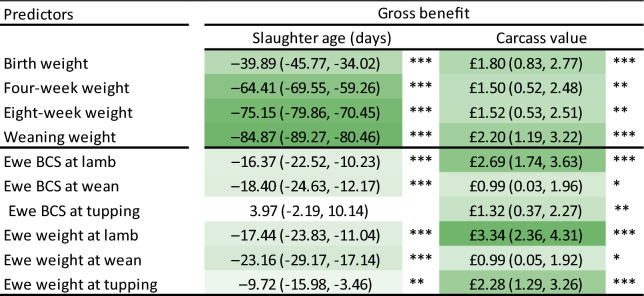
Gross information values of individual predictors.

Darker shades indicate higher information values.

Confidence intervals (95%) shown in parentheses.

Significance codes: ***p < 0.001; **p < 0.01; *p < 0.05.

**Figure 2:**
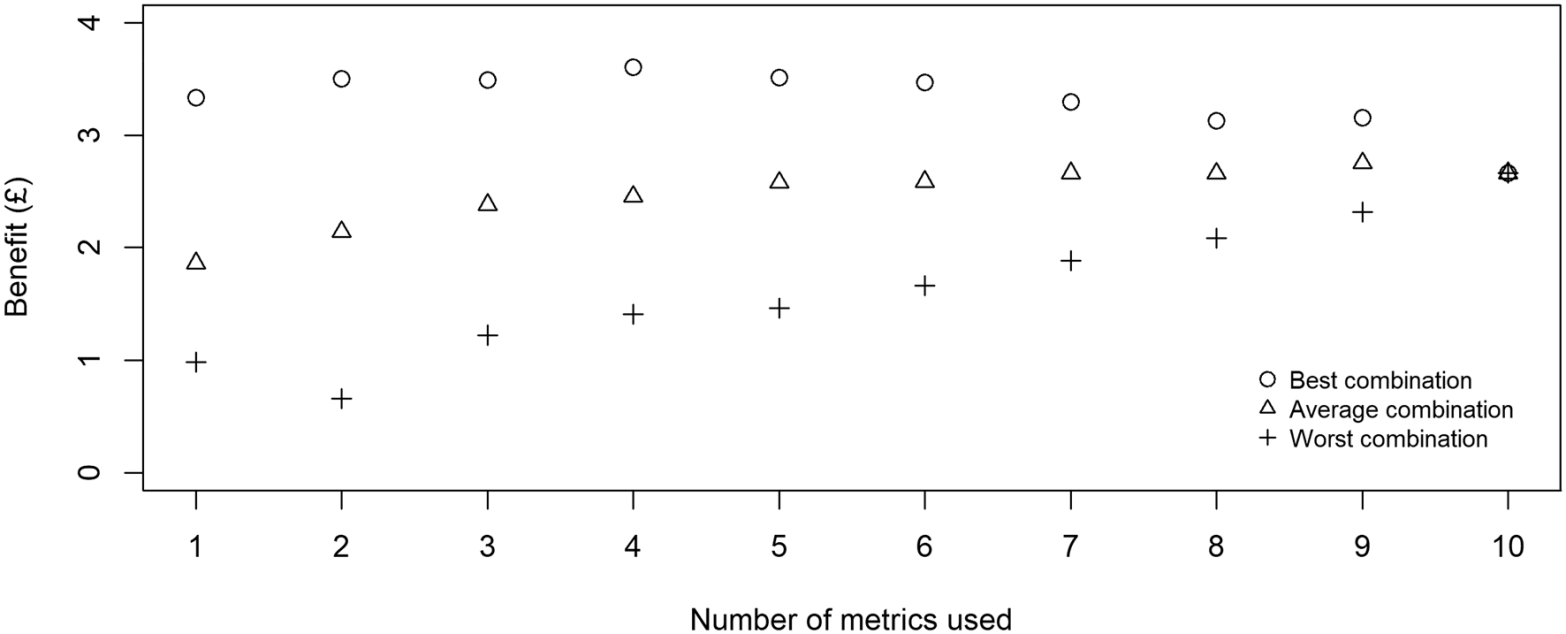
Combined gross information value of multiple predictors. A considerable variability in information value is observed even when the same number of predictors is used, demonstrating the importance of selecting key performance indicators based on quantitative evidence.

Figure 2 shows the combined benefits of multiple predictors under the best, average and worst combinations when different numbers of metrics are used. The gap in information value between the best and worst combinations was found to be pronounced, up to £2.84 under two predictors. This difference gradually reduced as more predictors were added until all 10 predictors were included (thus there is only one ‘combination’). Large differences were also observed between the best and average combinations of predictors, suggesting that predictors which are chosen randomly have substantially less information value than those selected on evidence.

Across all ‘best’ combinations (using 1–10 predictors), peak benefit of £3.61 was recorded under four predictors: ewe weight at lambing, ewe BCS at lambing, ewe BCS at tupping and lamb weight at birth. The inclusion of additional metrics beyond this point reduced the gross economic benefit regardless of the combination selected. The predictors contributing to high value combinations are identified in Table 3a, with ewe weight and BCS at lambing both consistently featured in this list. Ewe weight and BCS at weaning, on the other hand, are consistently observed in the lowest ranked combinations, whether used individually or in combination with other predictors (Table 3b).

**Table 3:**
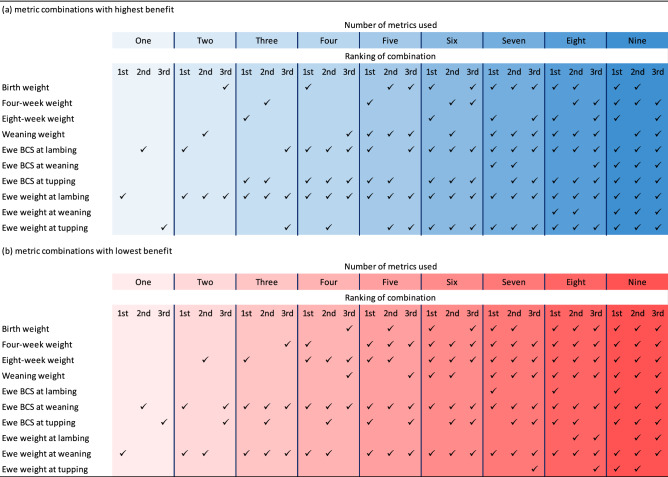
Predictors with highest and lowest values when used in combination with other predictors.

The results of sensitivity analysis suggested that the classification rule to define the high and low groups has a minimal impact on predictor rankings (Supplementary Tables S3 & S4). For the vast majority of cases, optimal combinations identified under the baseline method remained high-ranked under alternative rules (Supplementary Table S5), indicating that the findings reported above are not conditional on the inter-animal distribution intrinsic to the current dataset.

## Discussion

### Importance of ewe measurements

The above results indicated that the bodyweight and BCS of ewes have considerable economic importance as predictors of a farm’s performance. When ranked individually, the three most valuable predictors were associated with ewes rather than lambs (Table 2). The same tendency was also observed under composite rankings, where multiple predictors were combined to increase the overall information values (Table 3). These findings suggest that the impact of ewe health extends beyond pre-weaning lamb growth and affects farm profitability through multiple pathways. Thus, if one is forced to make a choice due to practical constraints, recording of ewe data should be prioritised over lamb data on commercial farms.

Compared to the high information values of ewe weight/BCS at lambing, the predictive power of ewe weight/BCS at weaning, while still present, was found to be somewhat muted. It is well established that ewe condition at lambing is associated with subsequent lamb growth rates, as it represents the energy reserves available for meeting the metabolic needs of lactation^50–52^. Contrarily, the exact purpose of ewe condition measurements at weaning — whether this is recommended to gain insight on the lambs’ growth prospect or to identify the ewe’s nutritional demand prior to the next tupping — has been rather ambiguous in the KPI literature. The present results suggest that this metric does not predict the current season’s lamb performance as accurately as ewe BCS at lambing. This is potentially due to the large variation across ewes, even amongst a single breed, in the amount of body reserves mobilised to meet the energy demand for lactation^53^.

Although ewe BCS at lambing appears to be most strongly linked to lamb growth and carcass value across all tested predictors, as stated this information is only meaningful if the cost of manipulating ewe BCS is outweighed by the subsequent economic benefit. Supplementing ewes with concentrate feeds during pregnancy is known to increase BCS at lambing^54^ and, in turn, improve lamb growth^55^; however, the benefit of using a high volume of concentrate feed for this purpose is unlikely to be large enough to justify the cost^56^ and can also invite a range of sustainability issues^9^. As an alternative strategy, a combined use of high-quality grass silage and concentrate feed, or deferred grazing post-lambing, is likely to be substantially more viable^57,58^.

Beyond a single season, lambs from ewes in better conditions finish faster and leave the farm earlier in the season, allowing a lower stocking rate for autumn grazing. This pasture surplus can then be used to improve ewe fertility through improved nutrition pre-mating^59^ or as supplemental feed during pregnancy^58^, creating a positive feedback loop across multiple seasons. A reduction in grazing pressure could also provide an environmental and ecological benefit, as grazing sheep at lower densities can increase the provision of ecosystem services, such as enhanced runoff water quality, plant productivity and carbon storage^60^. Alternatively, if less land area is required to produce a similar level of output through a shortened slaughter age, surplus land could be set aside for other purposes without compromising food security. Although much of the land used for sheep grazing in the world is marginal and often unsuitable for cultivation of human-edible crops^6,61^, afforestation of this surplus land would sequester carbon^62^ and rewilding of this land would facilitate the restoration of both biodiversity and ecosystem processes^63,64^. Both of these approaches can mitigate the environmental impact of agriculture and at the same time increase farm resilience against future external shocks, especially in relation to the future potential of carbon credits to support agroecological farming^65^.

### Cost of recording information

While our analyses demonstrated a positive gross economic benefit of recording information on the farm, gathering this information is seldom free of cost. On large commercial farms, labour cost is generally monetised. Even on traditional family farms where labour time is often not considered a tangible financial cost, labour saving can allow time to be devoted to other tasks and thus indirectly contributes to operational profitability^66^. As already discussed, sheep farms can take a wide variety of enterprise structures and, as such, care should be exercised to apply a particular cost assumption to draw general conclusions about the overall financial implications of on-farm measurements. Nevertheless, to assess the value of information in a holistic manner, the costs of both labour time and any necessary equipment must be considered.

To investigate the potential impact of these burdens on the results reported above, an auxiliary analysis was conducted to estimate the *net* information value of each individual predictor with respect to the resultant carcass value. Three cost scenarios were considered based on financial information from the NWFP: (1) equipment is purchased solely for predictor measurements; (2) equipment is newly purchased but its cost is shared between seasonal operational measurements and predictor measurements; and (3) equipment already exists and therefore recording only incurs labour cost (Table 4). As expected, the absolute value of net benefit was highly sensitive to the cost assumption. However, the relative benefit between predictors remained unchanged, indicating that the priority ranking complied from the *gross* information value is robust to the cost assumption adopted (Table 5).

**Table 4:** Cost scenarios used to estimate net information values.

Measurement	Equipment cost per lamb^†^	Labour cost per lamb^‡^	Total cost per lamb
**Scenario 1. Equipment is purchased solely for predictor measurements**			
Ewe weight*	£1.37	£0.30	£0.89
Ewe BCS*	£1.37	£0.35	£0.91
Lamb weight	£0.82	£0.30	£1.12
**Scenario 2. Equipment is newly purchased but its cost is shared with operational measurements (once a year)**			
Ewe weight*	£0.51	£0.30	£0.43
Ewe BCS*	£0.51	£0.35	£0.46
Lamb weight	£0.41	£0.30	£0.71
**Scenario 3. Equipment already exists and therefore recording only incurs labour cost**			
Ewe weight*	-	£0.30	£0.16
Ewe BCS*	-	£0.35	£0.19
Lamb weight	-	£0.30	£0.30

* Corrected for the average litter size (1.88).

^†^ Based on the following assumptions about capital costs and life cycles — SRS2 stick reader: £620.17 over 5 years. EziWeigh7i weighing head: £815.08 over 10 years. Border Software weigh crate: £2724 over 10 years. Handling system: £5395 over 30 years.

^‡^ Based on the following assumptions about labour requirements and wage rate — Weighing: 0.9 min per animal. BCS: 1.05 min per animal. Wage rate: £20 per hour or 0.33p per minute (covering two workers).

**Table 5:**
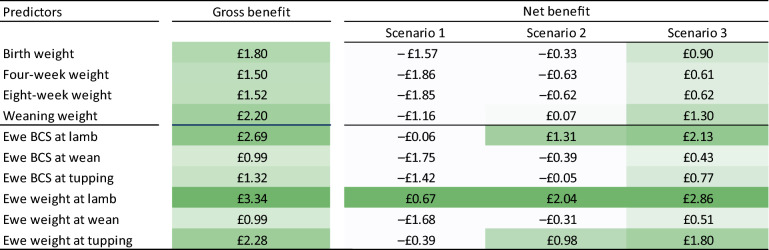
Net information values of individual predictors based on realised carcass value.

Darker shades indicate higher information values.

**Figure 3:**
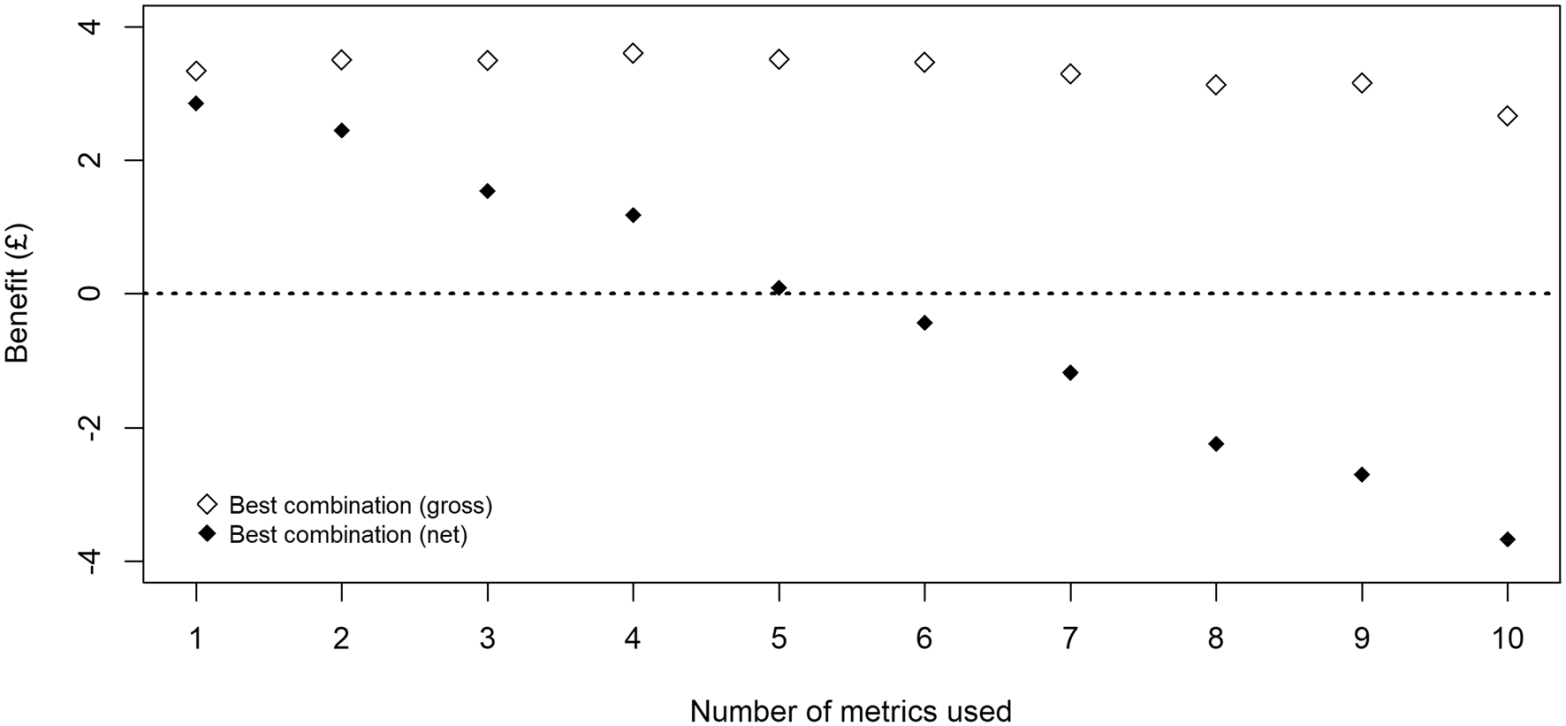
Gross and net information values of multiple predictors. Due to the flat shape of the gross curve, the net value linearly decreases as additional measurement costs are incurred.

When the third assumption was extended to composite rankings from multiple predictors, using six predictors or more resulted in a negative net information value (Fig. 3). This finding is driven by the combination of cumulative labour cost required to carry out additional measurements and the relatively small incremental gross benefit of using this information, the latter of which stems from a flat shape of the original response curve (Fig. 2). Between options with positive net information values, a single (non-composite) predictor (ewe weight at lambing) demonstrated the highest net value (£2.86), although the difference between this option and the best combination of two predictors (ewe weight and BCS at lambing, £2.45) was only marginal.

Further research is required, however, to investigate the production environment under which the above result of ‘you only need a single metric’ is applicable. As a research farm, the NWFP benefits from a higher allowance for labour input than most commercial farms, making good agricultural practices more easily implementable. In conjunction with a flock structure and management strategy which do not fluctuate between years, this contributes to a lower level of volatility in livestock productivity, and as a result less variation in ewe and lamb performance over time. The predictors used in this study therefore are likely to have a higher degree of correlation between them, which reduces the benefit of measuring additional predictors. Thus, on commercial farms that are less regimented and governed by managerial decisions more adaptive than prescriptive, the incremental benefit of using multiple predictors, thereby reducing statistical noise, may be more profound.

### Applicability in commercial settings

The analytical framework developed in this study provides an objective means to estimate the financial benefit of animal-level performance predictors. Practically speaking, however, the proposed method requires a certain degree of variability in both predictor and outcome variables; homogeneous animals reared under a single system cannot be differentiated. As the dataset used here originates from a research farm composed of three distinct grassland systems (permanent pasture, reseeded grass monoculture and reseeded legume/grass mix: see the Methods section), the validity of the framework within a single enterprise — the environment more resembling ordinary commercial farms — is worth evaluating. As such, the quantitative analysis described above was repeated separately for the three farmlets.

The results of this analysis were promising. For example, the most informative predictor for isolated use (ewe weight at lambing) was found to be worth £3.22, £3.26 and £3.99 across three systems, largely comparable to the value estimated for the full dataset (£3.34, Table 2). The best predictor combination for composite use (ewe weight at lambing, ewe BCS at lambing, ewe BCS at tupping and lamb weight at birth) were worth £3.52, £2.48 and £4.41, respectively, slightly fluctuated from the full dataset value (£3.49) but still all successfully (*p* < 0.05) differentiating the performance between the high and low groups as defined by predictor values. Given that the predictor variability *within a single farming system* is likely to be smaller on research farms than on commercial farms, the proposed method thus appears to be also suitable for data obtained outside an experimental environment.

Within individual farming systems, one possible use of the proposed framework is to pool data from multiple enterprises and develop a revised list of industry-recommended KPIs. As each KPI can now be accompanied by the potential economic value of the measurement, such a list may encourage more farmers to make an effort to obtain mid-season metrics to improve their production efficiency. Yet longer-term, the output from the current exercise should ideally become directly transformable to actionable benchmarks (trigger points) tailored for an individual farm. As a case in point, while our results clearly demonstrate the importance of maintaining ewe health during late pregnancy, this message on its own does not provide sufficient information to determine the exact timing at which interventions such as emergency supplementary feeding should be initiated.

As a step towards converting KPIs into actionable benchmarks, the relationship between the two highest-value predictors (ewe weight and BCS at lambing) and the carcass value of lambs was further investigated (Supplementary Tables S6 & S7). Rather than defining the high and low groups at a pre-determined proportion (e.g. top third and bottom third), the entire flock was split into two groups at multiple threshold values — in an increment of 1 kg for weight and 0.25 points for BCS. The information value calculated under each threshold value represents the maximum cost of intervention a farm would be willing to pay if animals in the low group are to be ‘transferred’ to the high group.

With ewe weight at lambing used as the predictor, the largest information value (£3.62) was observed when the threshold was set at 84 kg. However, the animals in the high group only accounted for 15% of the flock under this scenario, meaning that any ‘intervention’ would have to be applied almost blanketly across the whole farm. In addition to the practical challenges associated with a managerial change at this scale, this strategy is unlikely to prove financially viable, as the cost of intervention would be prohibitively high and the likelihood of successful intervention disproportionally low when performance targets are as ambitious. Ewe BCS at lambing, on the other hand, showed a more balanced split and an achievable target under the maximum information value (£2.40, 51% in the high group when the threshold is set at the BCS score of 3.25), and thus may provide an attractive alternative to bodyweight in this context^67^. Needless to say, full optimisation of intervention strategies would require detailed information on how animals respond to different forms of intervention, which is beyond the scope of the present study. Nevertheless, the proposed framework has two interrelated but separate pathways to facilitate evidence-based livestock farming, one through generic lists of recommended KPIs and another through more tailored decision support for individual farm management.

### Implications for the UK sheep sector

The results here demonstrated a high degree of variation in information value between different predictors, indicating that predictors selected through quantitative assessment are substantially more likely to have a positive impact on a farm’s profitability than those randomly or instinctively chosen. This information is particularly pertinent to the UK sheep sector today, as the country’s withdrawal from the European Union is predicted to have a detrimental impact on farm income when European-style direct payments are phased out from 2021^68,69^. Of all agricultural enterprises, sheep farms are predicted to be the worst affected, with some studies estimating that 70% of farms will be unprofitable once changes are in place^38^. Farms which are unable or unwilling to adapt to the new economic environment are likely to face bankruptcy, and many older farmers are expected to retire^70^.

The direct payments are to be succeeded by environmental land management schemes, which aim to improve the provision of ‘public money for public goods’ through environmental enhancement^71^. As this financial ‘support’ will only be provided in exchange for tangible provision of ecosystem services, it may lead to further fragmentation of the already stratified sheep sector^72^. In particular, sheep farms based in hill and upland areas, who have historically been the most reliant on agricultural subsidies^36^, will likely be pushed towards environmental land stewardship and away from sheep production^73,74^, rendering the findings of this study potentially less relevant^75,76^. Lowland sheep farms have generally been more productive and relatively less reliant on support payments, although in order to remain so in the absence of hill and upland farms, which often provide them with breeding units^72^, these farms will also need to make substantial improvements in profitability. These changes are likely to resemble those undergone by sheep farms in New Zealand following their agricultural transition in the late 1980s, which resulted in an increase in average farm size, reduction in labour input, identification of enterprise components contributing least to farm income and, ultimately, improvement in productivity^77–79^. Judging by this example, enhanced profitability is unlikely to be made without a detailed and accurate understanding of production processes and their contributions to the overall performance of the enterprise. The uptake of a more informed KPI decision support system, therefore, seems critical for UK sheep farms’ survival into the future.

### General discussion

The above analysis of UK sheep farms has provided a case exemplar of how the value of information can be defined and subsequently used to select the most useful predictors, or ‘key’ performance indicators, of which measurements should be prioritised. As stated above, the proposed framework is directly extendable to other livestock species and possibly beyond. Nonetheless, to effectively tailor the developed methodology to different farming enterprises, appropriate predictors, outcomes and cost assumptions must all be carefully considered.

For example, sheep in the UK are predominantly pasture-fed and undergo a yearly production cycle with a single crop of lambs that are valued according to their carcass weight and carcass quality^80^. Under this enterprise structure, the carcass value is arguably the most suitable outcome against which to assess the information value of predictors, as farm revenue is almost exclusively derived from this metric. However, for sectors operating under a less seasonal environment, for example indoor dairy and laying hen systems, outcome measures corresponding to the animal’s lifetime contribution to the enterprise may not be the most appropriate predictors, as they offer less opportunities for adaptive management^81,82^. In addition, the impact of measurement costs on the overall information value is likely to be smaller under these systems, especially if additional precision agriculture techniques are already in place to reduce labour requirements for information gathering^83,84^. Thus, the exact implementation process of the KPI selection framework will vary depending on the production system. Regardless, a holistic approach involving a wide range of factors contributing to farm profitability will remain essential to ensure the optimal system-wide information value.

Finally, while the role of animal-level KPIs in the improvement of overall farm efficiency has been clearly demonstrated in the present study, we acknowledge the complexity of livestock farming businesses beyond animal husbandry. Even the simplest form of farm enterprises face numerous non-livestock decisions on a daily basis^85^, to ensure, amongst others, soil health^86^, pasture growth^87,88^, and appropriate procurement and sales channels^89^. Each of these decisions can potentially be improved through additional information, of which collection and collation require labour time that competes against what is dedicated on animal husbandry. To this end, an extended framework to optimise the enterprise-wide information value of both livestock and non-livestock measurements is currently being developed.

## Supplementary Information

Supplementary Information.
