# Supplementary material for: Quantifying the value of on-farm measurements to inform the selection of key performance indicators for livestock production systems
Source: Sci Rep. 2021 Aug 19;11:16874. doi: 10.1038/s41598-021-96336-1 (PMC8377011; doi:10.1038/s41598-021-96336-1)
Supplement: Supplementary file 1 — Supplementary Information. [file 41598_2021_96336_MOESM1_ESM.pdf]

Quantifying the value of on-farm measurements to inform the selection of key performance indicators for livestock production systems

Andy Jones[1,2], Taro Takahashi[1,2] *, Hannah Fleming[1], Bruce Griffith[1], Paul Harris[1] and Michael Lee[3]

[1] *Rothamsted Research, North Wyke, Okehampton, Devon, EX20 2SB, UK*

[2] *University of Bristol, Langford, Somerset, BS40 5DU, UK*

[3] *Harper Adams University, Newport, Shropshire, TF10 8NB, UK*

* Corresponding author: taro.takahashi@rothamsted.ac.uk

Supplementary Material (8 pages)

Table S1 Correlation matrix between performance predictors.

Table S2 P-values for correlations between performance predictors.

Table S3 Predictors with high and low information values when used in combination with other predictors — under the quartile rule (25%/50%/25%) to define top and bottom groups

Table S4 Predictors with high and low information values when used in combination with other predictors — under the equal halves rule (50%/50%) to define top and bottom groups

Table S5 Rankings of high-value and low-value predictor combinations under alternative definitions of top and bottom groups

Table S6 Actionable benchmarks determined by ewe's weight at lambing

Table S7 Actionable benchmarks determined by ewe's BCS at lambing

Table S1. Correlation matrix between performance predictors

	BW	A4W	A8W	WW	BAL	BAW	BAT	WAL	WAW	WAT
BW	1									
A4W	0.696	1								
A8W	0.599	0.880	1							
WW	0.476	0.760	0.853	1						
BAL	0.106	0.271	0.225	0.192	1					
BAW	0.057	0.148	0.170	0.194	0.428	1				
BAT	-0.083	-0.123	-0.136	-0.080	0.189	0.252	1			
WAL	0.196	0.306	0.251	0.242	0.589	0.247	0.017	1		
WAW	0.151	0.193	0.176	0.249	0.317	0.594	0.137	0.639	1	
WAT	0.125	0.143	0.112	0.125	0.247	0.180	0.219	0.689	0.641	1

BW=lamb birth weight; A4W=adjusted lamb weight at four weeks; A8W=adjusted lamb weight at eight weeks; WW=lamb weight at weaning; BAL=ewe's body condition score at lambing; BAW=ewe's body condition score at weaning; BAT=ewe's body condition score at tupping; WAL=ewe's weight at lambing; WAW=ewe's weight at weaning; WAT=ewe's weight at tupping.

Table S2. *P*-values for correlations between performance predictors

	BW	A4W	A8W	WW	BAL	BAW	BAT	WAL	WAW	WAT
BW	0									
A4W	<0.001	0								
A8W	<0.001	<0.001	0							
WW	<0.001	<0.001	<0.001	0						
BAL	<0.001	<0.001	<0.001	<0.001	0					
BAW	0.073	<0.001	<0.001	<0.001	<0.001	0				
BAT	0.009	<0.001	<0.001	0.010	<0.001	<0.001	0			
WAL	<0.001	<0.001	<0.001	<0.001	<0.001	<0.001	0.528	0		
WAW	<0.001	<0.001	<0.001	<0.001	<0.001	<0.001	<0.001	<0.001	0	
WAT	<0.001	<0.001	<0.001	<0.001	<0.001	<0.001	<0.001	<0.001	<0.001	0

BW=lamb birth weight; A4W=adjusted lamb weight at four weeks; A8W=adjusted lamb weight at eight weeks; WW=lamb weight at weaning; BAL=ewe's body condition score at lambing; BAW=ewe's body condition score at weaning; BAT=ewe's body condition score at tupping; WAL=ewe's weight at lambing; WAW=ewe's weight at weaning; WAT=ewe's weight at tupping.

All values have been adjusted for multiple tests using the Holm method.

Table S3. Predictors with high and low information values when used in combination with other predictors — under the quartile rule (25%/50%/ 25%) to define top and bottom groups

(a) metric combinations with highest benefit

	Number of metrics used																										
	One			Two			Three			Four			Five			Six			Seven			Eight			Nine		
	Ranking of combination																										
	1st	2nd	3rd	1st	2nd	3rd	1st	2nd	3rd	1st	2nd	3rd	1st	2nd	3rd	1st	2nd	3rd	1st	2nd	3rd	1st	2nd	3rd	1st	2nd	3rd
Birth weight									✓					✓		✓	✓	✓			✓	✓	✓	✓	✓	✓	
Four-week weight								✓				✓	✓				✓		✓	✓	✓	✓	✓	✓	✓		✓
Eight-week weight										✓					✓	✓			✓		✓	✓	✓			✓	✓
Weaning weight					✓	✓	✓		✓		✓		✓		✓	✓			✓	✓	✓	✓	✓	✓	✓	✓	✓
Ewe BCS at lambing			✓	✓		✓	✓	✓		✓	✓	✓	✓	✓	✓	✓	✓	✓	✓	✓	✓	✓	✓	✓	✓	✓	✓
Ewe BCS at weaning																	✓			✓				✓	✓	✓	✓
Ewe BCS at tupping														✓				✓	✓	✓			✓	✓	✓	✓	✓
Ewe weight at lambing	✓			✓	✓		✓	✓	✓	✓	✓	✓	✓	✓	✓	✓	✓	✓	✓	✓	✓	✓	✓	✓	✓	✓	✓
Ewe weight at weaning																		✓				✓			✓	✓	✓
Ewe weight at tupping		✓								✓	✓	✓	✓	✓	✓	✓	✓	✓	✓	✓	✓	✓	✓	✓	✓	✓	✓

(b) metric combinations with lowest benefit

	Number of metrics used																										
	One			Two			Three			Four			Five			Six			Seven			Eight			Nine		
	Ranking of combination																										
	1st	2nd	3rd	1st	2nd	3rd	1st	2nd	3rd	1st	2nd	3rd	1st	2nd	3rd	1st	2nd	3rd	1st	2nd	3rd	1st	2nd	3rd	1st	2nd	3rd
Birth weight											✓					✓		✓	✓	✓	✓	✓	✓	✓	✓	✓	✓
Four-week weight						✓		✓	✓	✓			✓	✓	✓	✓	✓		✓	✓	✓	✓	✓	✓	✓	✓	✓
Eight-week weight						✓				✓	✓	✓	✓	✓	✓	✓	✓	✓	✓	✓	✓	✓	✓	✓	✓	✓	✓
Weaning weight													✓				✓	✓				✓	✓		✓	✓	✓
Ewe BCS at lambing																				✓	✓	✓	✓	✓	✓	✓	
Ewe BCS at weaning		✓		✓	✓		✓	✓	✓	✓	✓	✓	✓	✓	✓	✓	✓	✓	✓	✓	✓	✓	✓	✓	✓	✓	✓
Ewe BCS at tupping			✓		✓		✓		✓			✓		✓			✓	✓	✓		✓	✓		✓	✓	✓	✓
Ewe weight at lambing																✓								✓	✓		✓
Ewe weight at weaning	✓			✓			✓	✓		✓	✓	✓	✓	✓	✓	✓	✓	✓	✓	✓	✓	✓	✓	✓	✓	✓	✓
Ewe weight at tupping															✓				✓	✓			✓			✓	✓

Table S4. Predictors with high and low information values when used in combination with other predictors — under the equal half rule (50%/ 50%) to define top and bottom groups

(a) metric combinations with highest benefit

	Number of metrics used																										
	One			Two			Three			Four			Five			Six			Seven			Eight			Nine		
	Ranking of combination																										
	1st	2nd	3rd	1st	2nd	3rd	1st	2nd	3rd	1st	2nd	3rd	1st	2nd	3rd	1st	2nd	3rd	1st	2nd	3rd	1st	2nd	3rd	1st	2nd	3rd
Birth weight					✓			✓				✓	✓	✓		✓		✓	✓		✓	✓	✓	✓	✓	✓	✓
Four-week weight						✓											✓		✓	✓		✓	✓		✓	✓	✓
Eight-week weight							✓				✓				✓			✓		✓	✓		✓	✓	✓	✓	✓
Weaning weight			✓							✓			✓	✓		✓	✓		✓	✓	✓	✓		✓	✓	✓	✓
Ewe BCS at lambing	✓							✓	✓	✓	✓	✓	✓	✓	✓	✓	✓	✓	✓	✓	✓	✓	✓	✓	✓	✓	✓
Ewe BCS at weaning															✓		✓		✓			✓	✓	✓	✓		✓
Ewe BCS at tupping							✓		✓	✓	✓		✓	✓	✓	✓	✓	✓	✓	✓	✓	✓	✓	✓	✓	✓	✓
Ewe weight at lambing		✓		✓	✓	✓	✓	✓	✓	✓	✓	✓	✓		✓	✓	✓	✓	✓	✓	✓	✓	✓	✓	✓	✓	✓
Ewe weight at weaning																										✓	✓
Ewe weight at tupping				✓								✓		✓		✓		✓		✓	✓	✓	✓	✓	✓	✓	

(b) metric combinations with lowest benefit

	Number of metrics used																										
	One			Two			Three			Four			Five			Six			Seven			Eight			Nine		
	Ranking of combination																										
	1st	2nd	3rd	1st	2nd	3rd	1st	2nd	3rd	1st	2nd	3rd	1st	2nd	3rd	1st	2nd	3rd	1st	2nd	3rd	1st	2nd	3rd	1st	2nd	3rd
Birth weight														✓	✓	✓		✓	✓	✓	✓	✓	✓	✓	✓	✓	✓
Four-week weight									✓		✓	✓	✓		✓	✓	✓	✓		✓	✓	✓	✓		✓	✓	✓
Eight-week weight		✓		✓		✓		✓	✓	✓		✓	✓	✓		✓	✓	✓	✓	✓	✓	✓	✓	✓	✓	✓	✓
Weaning weight							✓			✓	✓		✓		✓	✓	✓	✓	✓	✓	✓	✓	✓	✓	✓	✓	✓
Ewe BCS at lambing																	✓		✓			✓			✓		✓
Ewe BCS at weaning	✓			✓	✓		✓	✓	✓	✓	✓	✓	✓	✓	✓	✓	✓		✓	✓		✓	✓	✓	✓	✓	✓
Ewe BCS at tupping																		✓			✓		✓	✓		✓	✓
Ewe weight at lambing																								✓	✓	✓	
Ewe weight at weaning			✓		✓	✓	✓	✓		✓	✓	✓	✓	✓	✓	✓	✓	✓	✓	✓	✓	✓	✓	✓	✓	✓	✓
Ewe weight at tupping														✓					✓	✓	✓	✓	✓	✓	✓	✓	✓

Table S5. Rankings of high-value and low-value predictor combinations under alternative definitions of top and bottom groups

(a) metric combinations with highest benefit under baseline analysis

	One			Two			Three			Four			Five			Six			Seven			Eight			Nine		
Baseline (thirds)	1	2	3	1	2	3	1	2	3	1	2	3	1	2	3	1	2	3	1	2	3	1	2	3	1	2	3
Quarters	1	3	2	1	2	5	11	4	10	23	24	13	25	38	5	12	19	15	17	37	22	19	10	25	7	1	3
Halves	2	1	4	6	7	2	1	6	5	13	57	1	21	13	5	3	4	5	70	8	3	14	16	4	7	6	5
Combinations*		10			45			120			210			252			210			120			45			10	

(b) metric combinations with lowest benefit under baseline analysis

	One			Two			Three			Four			Five			Six			Seven			Eight			Nine		
Baseline (thirds)	1	2	3	1	2	3	1	2	3	1	2	3	1	2	3	1	2	3	1	2	3	1	2	3	1	2	3
Quarters	1	2	3	1	15	2	6	1	2	1	3	50	2	8	5	14	2	6	7	4	13	1	7	16	2	3	1
Halves	3	1	4	2	3	7	2	18	8	3	20	25	45	6	35	1	17	33	7	8	41	13	28	5	3	2	8
Combinations*		10			45			120			210			252			210			120			45			10	

* Unique patterns available under each number of metrics

Table S6. Actionable benchmarks determined by ewe's weight at lambing

Top group, >= 'X' kg	Carcass value of top group	Carcass value of bottom group	Difference	Proportion requiring intervention
86	£77.22	£73.98	£3.23	90%
85	£77.17	£73.90	£3.27	87%
84	£77.39	£73.77	£3.62	85%
83	£77.06	£73.76	£3.30	83%
82	£76.99	£73.69	£3.30	81%
81	£76.94	£73.58	£3.36	78%
80	£76.81	£73.52	£3.29	76%
79	£76.93	£73.35	£3.59	73%
78	£76.75	£73.31	£3.44	71%
77	£76.58	£73.25	£3.33	68%
76	£76.02	£73.35	£2.67	64%
75	£75.84	£73.32	£2.52	60%
74	£75.68	£73.31	£2.37	58%
73	£75.56	£73.18	£2.39	52%
72	£75.33	£73.19	£2.14	48%
71	£75.22	£73.17	£2.05	44%
70	£75.06	£73.22	£1.85	40%
69	£74.96	£73.05	£1.92	34%
68	£75.00	£72.72	£2.27	30%
67	£74.87	£72.74	£2.13	26%
66	£74.81	£72.55	£2.26	22%
65	£74.71	£72.59	£2.12	19%
64	£74.59	£72.84	£1.75	16%
63	£74.54	£72.82	£1.73	13%
62	£74.54	£72.54	£2.00	11%
61	£74.50	£72.44	£2.06	9%

Table S7. Actionable benchmarks determined by ewe's BCS at lambing

Top group, >= 'X' BCS	Carcass value of top group	Carcass value of bottom group	Difference	Proportion requiring intervention
4	£76.11	£74.23	£1.88	95%
3.75	£76.42	£74.04	£2.37	88%
3.5	£75.77	£73.59	£2.18	67%
3.25	£75.53	£73.13	£2.40	51%
3	£74.69	£73.25	£1.44	26%
2.75	£74.59	£73.28	£1.31	21%
2.5	£74.35	£73.99	£0.36	10%
2.25	£74.29	£74.69	-£0.40	6%
2	£74.33	£72.07	£2.27	1%
